# Supplementary material for: Prognostic value of FOXA1 in estrogen receptor-negative breast cancer: A systematic review and meta-analysis
Source: PLoS One. 2025 Oct 21;20(10):e0332516. doi: 10.1371/journal.pone.0332516 (PMC12539746; doi:10.1371/journal.pone.0332516)
Supplement: S1 Table — (PDF) [file pone.0332516.s001.pdf]

S1 Table: Database Search Term

Electronic Search Report No. 1	
**Search type**	New
**Databases**	MEDLINE
**Platform**	PUBMED
**Search date**	03/07/2025
**Search date range**	Without Restriction
**Language restrictions**	None
**Other limits**	None
**Search strategy**	((((((((((Breast cancer[Title/Abstract]) OR (Breast neoplasm[Title/Abstract])) OR (Breast Tumor[Title/Abstract])) OR (Cancer of breast[Title/Abstract])) OR (Carcinoma Breast[Title/Abstract])) OR (Breast Carcinomas[Title/Abstract])) OR (Tumor Breast[Title/Abstract])) OR (Breast carcinoma[Title/Abstract])) OR (mammary cancer[Title/Abstract])) OR (breast malignancy[Title/Abstract])) AND ((((((((FOXA1) OR (HNF-3A protein, human)) OR (forkhead box A1 protein, human)) OR (hepatocyte nuclear factor 3alpha)) OR (forkhead box A1 protein)) OR (FOXA1 protein)) OR (forkhead box protein A1)) OR (HNF 3 alpha))) AND ((((((((((Prognosis[Title/Abstract]) OR (Prognostic Factors[Title/Abstract])) OR (Neoplasm Invasiveness[Title/Abstract])) OR (Invasion, Neoplasm[Title/Abstract])) OR (Invasiveness, Neoplasm[Title/Abstract])) OR (Disease Free Survival[Title/Abstract])) OR (Survival, Disease-Free[Title/Abstract])) OR (cancer prognosis[Title/Abstract])) OR (disease free survival[Title/Abstract])) OR (DFS (disease free survival[Title/Abstract]))) OR (disease-free survival[Title/Abstract]))

Electronic Search Report No. 2	
**Search type**	New
**Databases**	EMBASE
**Platform**	Elsevier
**Search date**	03/07/2025
**Search date range**	Without Restriction
**Language restrictions**	None
**Other limits**	None
**Search strategy**	('breast cancer'/exp OR 'ca breast' OR 'breast cancer' OR 'breast gland cancer' OR 'breast gland neoplasm' OR 'breast malignancies' OR 'breast malignancy' OR 'breast tumor malignant' OR 'cancer in the mammary gland' OR 'cancer of the breast' OR 'cancer of the mammary gland' OR 'cancer, breast' OR 'malignancies of the breast' OR 'malignancy of the breast' OR 'malignant breast neoplasm' OR 'malignant breast tumor' OR 'malignant neoplasm of the breast' OR 'malignant tumor of the breast' OR 'mamma cancer' OR 'mammary cancer' OR 'mammary gland cancer' OR 'mammary gland malignancy' OR 'mammary malignancies' OR 'mammary malignancy') AND ('hepatocyte nuclear factor

	3alpha'/exp OR 'foxa1 protein' OR 'hnf 3alpha' OR 'hnf3alpha' OR 'forkhead box a1 protein' OR 'forkhead box protein a1' OR 'hepatocyte nuclear factor 3 alpha' OR 'hepatocyte nuclear factor 3-alpha' OR 'hepatocyte nuclear factor 3alpha' OR 'protein foxa1') AND ('disease free survival'/exp OR 'dfs (disease free survival)' OR 'disease free survival' OR 'disease-free survival' OR 'prognosis'/exp OR 'survival'/exp OR 'survival')

**Electronic Search Report No. 3**	
**Search type**	New
**Databases**	SCOPUS
**Platform**	SCOPUS
**Search date**	03/07/2025
**Search date range**	Without Restriction
**Language restrictions**	None
**Other limits**	None
**Search strategy**	(TITLE-ABS-KEY (prognosis OR "Prognostic Factors" OR "cancer prognosis" OR "Neoplasm Invasiveness" OR "disease free survival" OR "DFS (disease free survival)" OR "disease-free survival" OR "Survival, Disease-Free" OR survival)) AND ((TITLE-ABS-KEY ("Breast cancer" OR "Breast carcinoma" OR "Breast tumor" OR "breast malignancy" OR "mammary cancer")) AND (TITLE-ABS-KEY ("hepatocyte nuclear factor 3 alpha" OR "forkhead box A1 protein" OR "FOXA1 protein" OR "forkhead box protein A1" OR "HNF 3alpha")))

**Electronic Search Report No. 4**	
**Search type**	New
**Databases**	GOOGLE SCHOLAR
**Platform**	GOOGLE SCHOLAR
**Search date**	03/07/2025
**Search date range**	Without Restriction
**Language restrictions**	None
**Other limits**	None
**Search strategy**	"breast cancer" OR "breast neoplasm" + "FOXA1" OR "HNF-3A protein, human" + "prognosis" OR "survival"

**Electronic Search Report No. 5**	
**Search type**	New
**Databases**	Lilacs
**Platform**	BVS
**Search date**	03/07/2025
**Search date range**	Without Restriction
**Language restrictions**	None

**Other limits**	None
**Search strategy**	((breast cancer) OR (cancer de seno)) AND ((foxa1) OR (proteina humana hnf-3a)) AND ((survival) OR (pronóstico)) AND (db:("MEDLINE"))
