# Supplementary material for: Prognostic value of FOXA1 in estrogen receptor-negative breast cancer: A systematic review and meta-analysis
Source: PLoS One. 2025 Oct 21;20(10):e0332516. doi: 10.1371/journal.pone.0332516 (PMC12539746; doi:10.1371/journal.pone.0332516)
Supplement: S2 Table — (PDF) [file pone.0332516.s002.pdf]

S2 Table: List of excluded items and reason for exclusion

Author-Year	Include/Exclude	Reason for Exclusion
Xu, 2017	Exclude	No ER-negative
Badve, 2007	Exclude	No ER-negative
Thorat, 2008	Exclude	No ER-negative
Badve, 2006	Exclude	Meeting
Salta, 2018	Exclude	No ER-negative
Guiu, 2015	Exclude	No Outcome
Gucalp, 2014	Exclude	Meeting
Jiang, 2016	Exclude	No FOXA1 alone
Jacquemier, 2009	Exclude	No Outcome
Hisamatsu, 2011	Exclude	Meeting
Yang, 2020	Exclude	Meeting
Ademuyiwa, 2010	Exclude	No Outcome
Liu. 2010	Exclude	No ER-negative
Dieci, 2019	Exclude	No FOXA1 alone
Kong, 2013	Exclude	No FOXA1 alone
Dedina, 2017	Exclude	Meeting
Badve, 2009	Exclude	Meeting
Aleskandarany, 2016	Exclude	No FOXA1 alone
Tokunaga, 2010	Exclude	Meeting
Zhang, 2016	Exclude	Variant
Omata, 2018	Exclude	No FOXA1 alone
Abelzaher, 2020	Exclude	No Outcome
Bobrie, 2022	Exclude	No FOXA1 alone
Abduljabbar, 2015	Exclude	No FOXA1 alone
Sheta, 2021	Exclude	Other cancer
Rangel, 2018	Exclude	No ER-negative
Horimoto, 2015	Exclude	No ER-negative
Schrijver, 2018	Exclude	No ER-negative
Tokunaga, 2012	Exclude	No ER-negative
Liu, 2010	Exclude	Review
Liu, 2012	Exclude	No FOXA1 alone
Humphries, 2017	Exclude	No ER-negative
MacÃas-GarcÃa, 2016	Exclude	No ER-negative
Ciriello, 2015	Exclude	No FOXA1 alone
Richard, 2020	Exclude	No Outcome
Li, 2015	Exclude	No FOXA1 alone
Badve, 2010	Exclude	No FOXA1 alone
Jacot, 2023	Exclude	Meeting
Graeser, 2023	Exclude	No ER-negative

Asleh, 2022	Exclude	No Outcome
Kumegawa, 2022	Exclude	No Outcome
Guiu, 2018	Exclude	No FOXA1 alone
Kometova, 2022	Exclude	Meeting
Lin, 2021	Exclude	No FOXA1 alone
BoissiÃ¨re-Michot, 2021	Exclude	No FOXA1 alone
Yu, 2021	Exclude	Meeting
Velimirovic, 2021	Exclude	Meeting
Bremer, 2020	Exclude	Meeting
Byun, 2020	Exclude	No ER-negative
GunnarsdÃ³ttir, 2020	Exclude	No outcome
Osako, 2020	Exclude	No ER-negative
Cohen, 2020	Exclude	Meeting
Bremer, 2020	Exclude	Meeting
Wadsten, 2019	Exclude	Meeting
Bremer, 2019	Exclude	Meeting
Nyqvist, 2019	Exclude	Meeting
Pascual, 2019	Exclude	Meeting
McNamara, 2018	Exclude	No FOXA1 alone
WÃ¤rnberg, 2018	Exclude	Meeting
Lavigne, 2018	Exclude	No FOXA1 alone
Joseph, 2017	Exclude	Meeting
Lauttia, 2017	Exclude	Meeting
Desmedt, 2016	Exclude	No Outcome
Lehmann-Che, 2013	Exclude	No FOXA1 alone
Kobayashi, 2013	Exclude	No ER-negative
Jaremko, 2009	Exclude	Meeting
Liu, 2022	Exclude	DataBase
Jacot, 2021	Exclude	No FOXA1 alone
Mehta, 2011	Exclude	No ER-negative
Severson, 2018	Exclude	No FOXA1 alone
Habashy, 2008	Exclude	No ER-negative
Hisamatsu, 2012	Exclude	No ER-negative
Ijichi, 2012	Exclude	No ER-negative
Kawase, 2015	Exclude	No ER-negative
Tanaka, 2017	Exclude	No Outcome
Horimoto, 2020	Exclude	No ER-negative
Govindan, 2020	Exclude	No FOXA1 alone
Hosoda, 2014	Exclude	No ER-negative
Mohsin, 2022	Exclude	Review
Li, 2022	Exclude	No FOXA1 alone

Joseph, 2019	Exclude	No FOXA1 alone
Yi, 2020	Exclude	No FOXA1 alone
Kutasovic, 2019	Exclude	No FOXA1 alone
Abdelhafiz, 2021	Exclude	No Outcome
Petrelli, 2020	Exclude	No Outcome
Servetto, 2021	Exclude	No Outcome
Byun, 2020	Exclude	Duplicaded
Gawrzak, 2018	Exclude	No FOXA1 alone
Chenpu Xu, 2015	Exclude	No ER-negative
Frietze, 2014	Exclude	No FOXA1 alone
Al-Bedairy, 2021	Exclude	No Outcome
BenAyed-Guerfali, 2019	Exclude	No ER-negative
