# Supplementary material for: Prognostic value of FOXA1 in estrogen receptor-negative breast cancer: A systematic review and meta-analysis
Source: PLoS One. 2025 Oct 21;20(10):e0332516. doi: 10.1371/journal.pone.0332516 (PMC12539746; doi:10.1371/journal.pone.0332516)

Supporting Figure 1: Sensitivity analysis. Forest plot of Disease-free survival as a function of FOXA1 expression in estrogen receptor-negative breast cancer CI: Confidence interval. Excluded.

Study or Subgroup	log[Hazard Ratio]	SE	Weight	Hazard Ratio IV, Random, 95% CI
Chen 2021 (271-285 H-S)	-0.9002	0.2949	14.1%	0.41 [0.23, 0.72]
Chen 2021 (285 H-S)	-0.4187	0.2345	17.4%	0.66 [0.42, 1.04]
Dai 2019 (40 H-S)	-0.1222	0.1276	24.2%	0.88 [0.69, 1.14]
Hisamatsu 2015 (71.7%)	-1.0188	0.4019	9.8%	0.36 [0.16, 0.79]
Jing 2019 (High)	-0.077	0.1103	25.2%	0.93 [0.75, 1.15]
Zhou 2023 (High)	-0.9555	0.4155	9.4%	0.38 [0.17, 0.87]
**Total (95% CI)**			**100.0%**	**0.65 [0.48, 0.87]**

Heterogeneity: $Tau^2 = 0.08$; $Chi^2 = 15.35$, df = 5 (P = 0.009); $I^2 = 67\%$
Test for overall effect: Z = 2.83 (P = 0.005)

Hazard Ratio
IV, Random, 95% CI

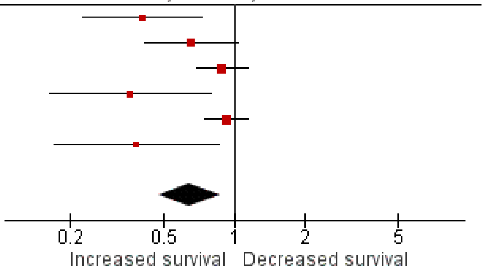

Supplement: S1 Fig — Forest plot of Disease-free survival as a function of FOXA1 expression in estrogen receptor-negative breast cancer CI: Confidence interval. Excluded. (PDF) [file pone.0332516.s004.pdf]
