# Supplementary material for: Prognostic value of FOXA1 in estrogen receptor-negative breast cancer: A systematic review and meta-analysis
Source: PLoS One. 2025 Oct 21;20(10):e0332516. doi: 10.1371/journal.pone.0332516 (PMC12539746; doi:10.1371/journal.pone.0332516)
Supplement: Attachment — Submitted filename: Response_to_reviewers_auresp_2.docx [file pone.0332516.s007.docx]

**Response to Reviewer**

PONE-D-25-39720

Prognostic value of FOXA1 in breast cancer estrogen receptor negative: A systematic review and meta-analysis

PLOS ONE

Dear Dr. Yan Xu, Ph.D.,

Academic Editor,

PLOS ONE

We thank the reviewer for their thorough and constructive feedback. We appreciate the time and expertise dedicated to reviewing our manuscript. All comments have been addressed point-by-point below, and we believe the suggested revisions have significantly improved the quality and clarity of our paper.

Is the manuscript presented in an intelligible fashion and written in standard English? **Reviewer #3: No**

**Response:** The manuscript was reviewed, and paragraph-by-paragraph corrections were made using the Grammarly Pro tool. During this process, the title was modified, and the relevant changes were highlighted.

Old title: Prognostic value of FOXA1 in breast cancer estrogen receptor negative: A systematic review and meta-analysis

New title: Prognostic Value of FOXA1 in Estrogen Receptor-Negative Breast Cancer: A Systematic Review and Meta-Analysis.

**Comment Reviewer #3:** In the abstract please revise the sentence stating treatment increases the risk of recurrence.

**Response:** In the abstract, the phrase "treatment increases the risk of recurrence" was changed to a more precise and scientifically correct phrase: "recurrence rates remain high despite current therapies for the disease."

**Comment Reviewer #3** Figure 1: fix the n= formatting in the excluded studies box for consistency on each line.

**Response:** We appreciate your attention to detail, the format of n= in Figure 1: has been corrected.

**Comment Reviewer #3:** In the paragraph about the risks of studies, say 6 out of 7 rather (or as well as) than 88%. And the same for the other percentages.

Response: As requested by the reviewer, the risk percentages in the indicated paragraph the risks of studies have been revised to reflect the actual number of articles.

**Comment Reviewer #3** Table 2: have the appropriate use of . For decimals for this audience (not ,)

**Response**: We thank the reviewer for their attention to detail. The respective corrections have been made in Table 2, and the changes have been highlighted for ease of review.

**Comment Reviewer #3:** Below figure 3 hazard rather than hazzard.

Response: We thank the reviewer for pointing out this typographical error. The misspelling of 'hazard' has been corrected in the text below Figure 3.
